# Successful continuous nivolumab therapy for metastatic non‐small cell lung cancer after local treatment of oligometastatic lesions

**DOI:** 10.1111/1759-7714.13539

**Published:** 2020-06-21

**Authors:** Satoshi Tobita, Yuhei Kinehara, Yoshio Tamura, Hiroyuki Kurebe, Ryusuke Ninomiya, Yoshihiko Utsu, Satoshi Kohmo, Bunzo Sato, Kenichi Nagai, Shintaro Maruoka, Ryu Jokoji, Shohei Koyama, Isao Tachibana

**Affiliations:** ^1^ Department of Medicine Nippon Life Hospital Osaka Japan; ^2^ Department of Gastrointestinal Surgery Nippon Life Hospital Osaka Japan; ^3^ Department of Radiotherapy Nippon Life Hospital Osaka Japan; ^4^ Department of Pathology Nippon Life Hospital Osaka Japan; ^5^ Department of Respiratory Medicine and Clinical Immunology Osaka University Graduate School of Medicine Osaka Japan

**Keywords:** Adaptive resistance, immune checkpoint inhibitors, local treatment, lymphocyte‐activation gene 3, T cell immunoglobulin and mucin domain‐containing protein 3

## Abstract

The patient in this report was a 57‐year‐old man with metastatic non‐small cell lung cancer (NSCLC). After no response to two lines of systemic chemotherapy, he was treated with nivolumab as third‐line therapy, which resulted in a partial response. After 17 months of nivolumab treatment, he developed bone metastasis in his left femur which was treated with radiation therapy. Nivolumab was restarted after radiation therapy. Four months after radiation therapy, he developed another metastatic lesion in the small intestine which was surgically resected. Because there were no recurrent NSCLC lesions after surgical resection, nivolumab was restarted again. At 18 months after surgery, there were no recurrent NSCLC lesions. Immunohistochemical analysis of peritumoral T lymphocytes showed higher expression of T cell immunoglobulin and mucin domain‐containing protein 3 (TIM‐3) and lymphocyte activation gene 3 (LAG‐3) in recurrent lesions of bone and small intestine than in primary lesions. Upregulation of TIM‐3 and LAG‐3 could be associated with mechanisms of adaptive resistance to nivolumab in this case. Here, we report a successful case of continued nivolumab therapy with remission after local treatments consisting of radiation therapy and surgical resection for oligometastases. Continuation of immune checkpoint inhibitor (ICI) treatment may be worth considering if oligometastases can be controlled.

**Key points:**

Significant findings of the study

We report a successful case of continued nivolumab treatment with remission after local treatment (radiation therapy and surgical resection) for oligometastases.

What this study adds

Upregulation of T cell immunoglobulin and mucin domain‐containing protein 3 and lymphocyte‐activation gene 3 could be associated with mechanisms of adaptive resistance to nivolumab.

## Graphical abstract

We report the first successful case of continued nivolumab treatment with remission after local treatment (radiation therapy and surgical resection) for oligometastases. The upregulation of T‐cell immunoglobulin and mucin domain‐containing protein 3 (TIM‐3) and lymphocyte‐activation gene 3 (LAG‐3) could be associated with mechanisms of adaptive resistance to nivolumab in this case.

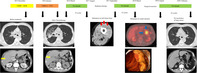

## Introduction

Anti‐programmed cell death 1 (PD‐1) monoclonal antibodies (mAbs) and anti‐programmed death‐ligand 1 (PD‐L1) mAbs counteract molecule‐mediated immunosuppressive signals. They have become key immune checkpoint inhibitors (ICIs).^1^, ^2^, ^3^ Combinations of conventional chemotherapeutic agents and ICIs have resulted in significantly longer overall and progression‐free survival than chemotherapy alone in many patients with previously untreated metastatic non‐small cell lung cancer (NSCLC).^4^, ^5^, ^6^ However, some patients who initially had a response have subsequently experienced relapse with resistance to this class of drugs after long‐term treatment.^7^, ^8^ It is unclear whether local therapy and continuation of ICI therapy could result in prolonged benefit for patients with adaptive resistance. Here, we report a rare case in which a patient achieved remission after local treatment for oligometastases consisting of both radiation therapy and surgical resection and was able to continue ICI treatment.

## Case report

**Figure 1:**
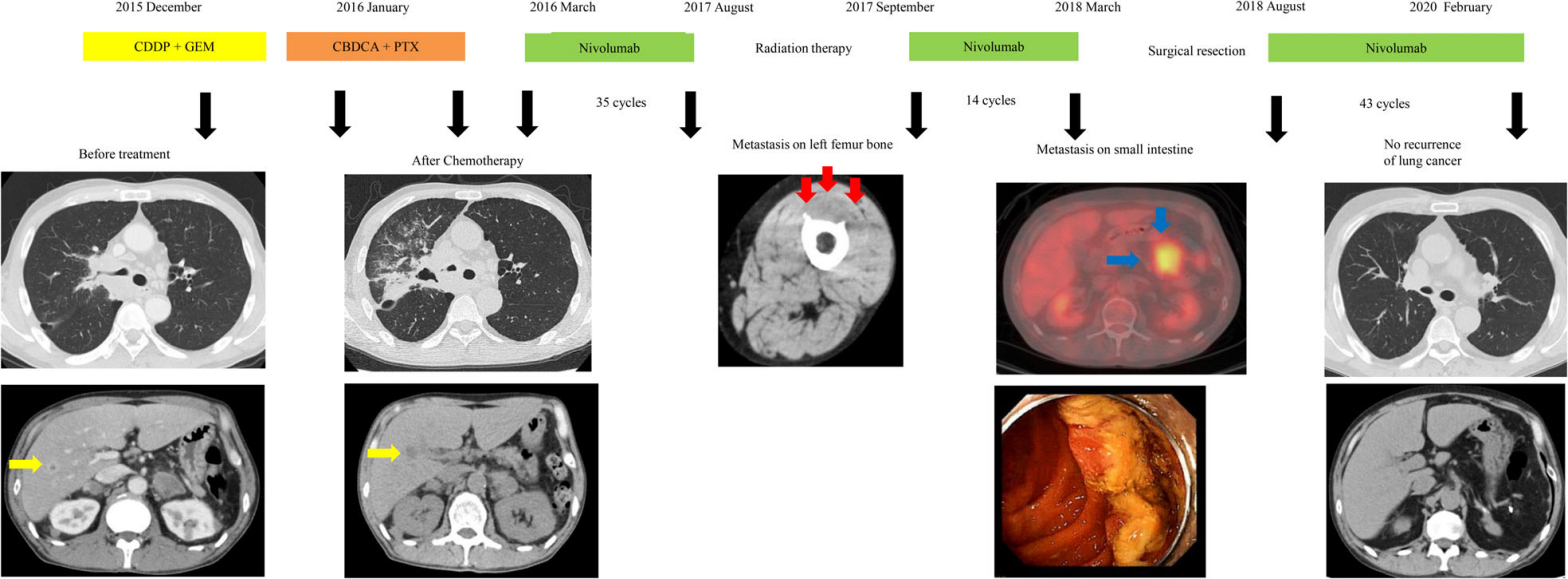
Clinical course. The yellow arrows indicate liver metastases. The red arrows indicate metastases in the left femur. The blue arrows indicate intestinal metastases. CDDP, cisplatin; GEM, gemcitabine; CBDCA, carboplatin; PTX, paclitaxel.

The patient was a 57‐year‐old man with T4N3M1c NSCLC. One cycle of cisplatin plus gemcitabine resulted in progressive disease (PD). Subsequently, two cycles of carboplatin plus paclitaxel resulted in PD. He was treated with nivolumab as third‐line therapy. When a partial response was confirmed, nivolumab was continued (Fig 1).

After 35 cycles of nivolumab, the patient developed bone metastasis in the left femur and received radiation therapy. Although he continued to be treated with nivolumab after radiation therapy, he developed another metastatic lesion in the small intestine after 49 additional cycles. He underwent surgical resection of the small intestine. Because there were no recurrent NSCLC lesions after surgical resection, treatment with nivolumab was reinitiated. At 18 months after surgery, whole‐body computed tomography (CT) showed no recurrence of lung cancer (Fig 1).

**Figure 2:**
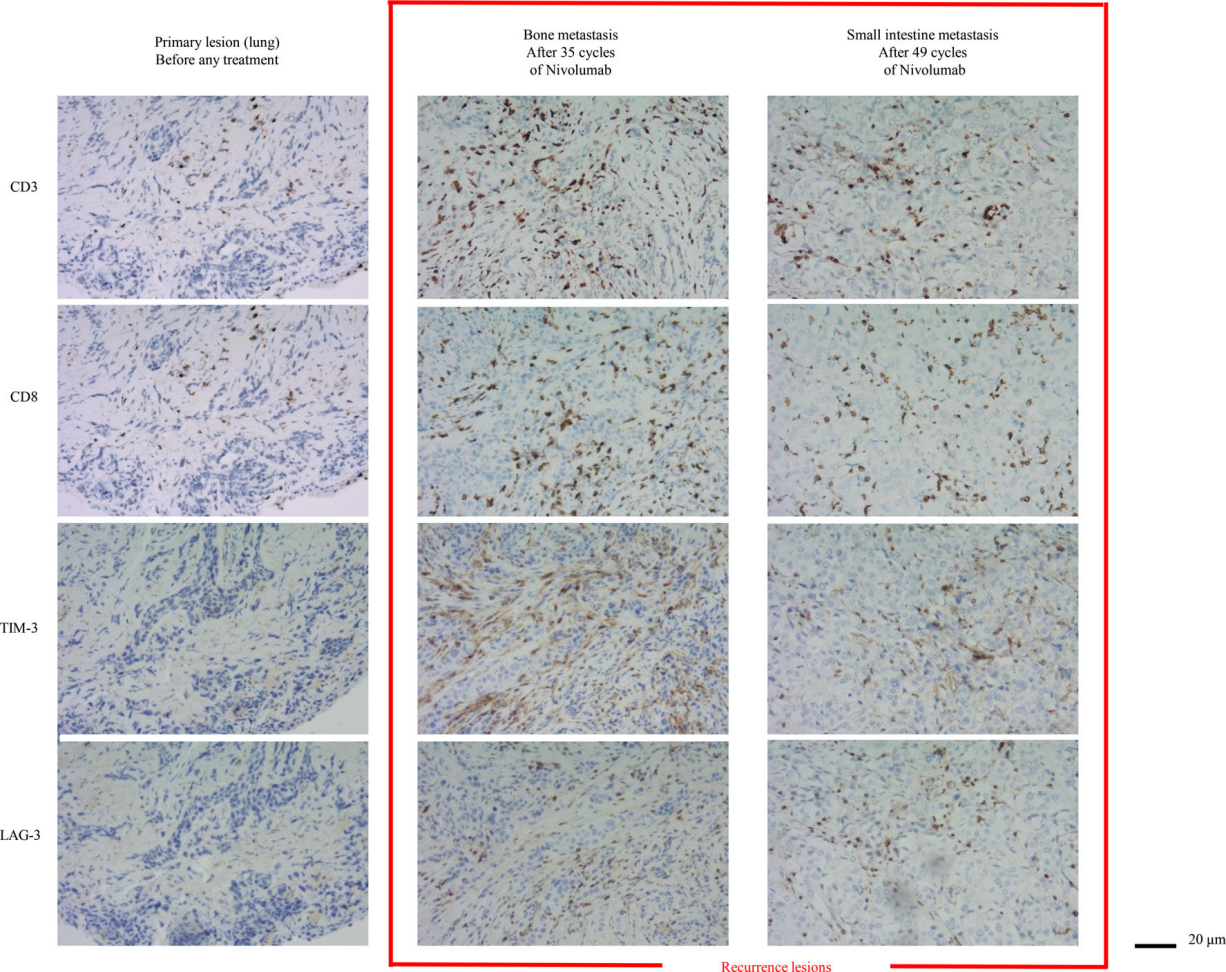
Immunohistochemical staining. Immunohistochemical staining for TIM‐3 and LAG‐3 in the primary lesion and recurrent lesions in the left femur and small intestine. TIM‐3, T cell immunoglobulin and mucin domain‐containing protein 3; LAG‐3, lymphocyte‐activation gene 3.

We analyzed the primary tumor and recurrent tumor with immunohistochemical staining. Peritumoral T lymphocytes had higher T cell immunoglobulin and mucin domain‐containing protein 3 (TIM‐3; D5D5R, 1:400, Cell Signaling Technology, Danvers, MA) and lymphocyte‐activation gene 3 (LAG‐3; D2G4O, 1:200, Cell Signaling Technology,) expression in recurrent lesions that developed during treatment with nivolumab than in primary lesions before any anticancer treatment (Fig 2).

## Discussion

We encountered a patient with oligometastases in whom remission was achieved after local treatment consisting of both radiation therapy and surgical resection. A retrospective study showed treatment with ICIs after progression is associated with improved survival in patients with NSCLC.^9^ Another retrospective study showed that local therapy for oligometastases and continuation of ICIs could result in prolonged benefit.^10^, ^11^ However, these studies were small and retrospective so it was not clear whether local therapy and continuation of ICI therapy could result in prolonged benefit for patients with adaptive resistance. We report a successful case of remission after local treatment for oligometastases consisting of both of radiation therapy and surgical resection; disease control was possible with continued nivolumab treatment. This case suggests that continuing both local treatment and ICIs is worth considering if oligometastases are locally controllable with treatments such as radiation therapy or surgical resection.

The mechanisms of resistance to ICI therapy are not yet well characterized, but likely involve multiple factors. It has been reported that loss of beta 2 microglobulin prevents major histocompatibility complex‐class I presentation of tumor antigens, loss of function *JAK1* and *JAK2* mutations results in insensitivity to interferon gamma, and longitudinal mutant neoantigen loss compromises immune recognition.^11^, ^12^, ^13^ Furthermore, the presence of parallel immune inhibitory pathways and loss of antigen presentation in cancer cells might be common mechanisms of resistance.^14^ Co‐expression of multiple T cell inhibitory receptors, including PD‐1, cytotoxic T lymphocytes antigen 4, TIM‐3, and LAG‐3 in activated or exhausted T cells suggests that parallel inhibitory pathways may mediate resistance to ICI therapy.^8^, ^11^ In this case, peritumoral T lymphocytes had higher TIM‐3 and LAG‐3 expression in recurrent lesions that developed during treatment with nivolumab than in primary lesions before any anticancer treatment. In this case, the upregulation of TIM‐3 and LAG‐3 might be associated with mechanisms of adaptive resistance to nivolumab. Because the continuation of nivolumab could have induced oligometastases of the small intestine after radiation therapy in this patient, combination therapy with an anti–PD‐1 antibody and anti‐TIM‐3 or LAG‐3 neutralizing antibodies may represent a promising strategy for overcoming adaptive resistance.

In conclusion, we encountered a case in which a patient with oligometastases achieved remission after local treatment consisting of radiation therapy and surgical resection. It may be worth considering continuing ICI treatment if there are no recurrent lesions after local treatment for oligometastases.

## Disclosure

The authors declare no conflicts of interest in association with the present study.

## References

[1] Rittmeyer A , Barlesi F , Waterkamp D *et al* Atezolizumab versus docetaxel in patients with previously treated non‐small‐cell lung cancer (OAK): A phase 3, open‐label, multicentre randomised controlled trial. Lancet 2017; 389: 255–65. 27979383 10.1016/S0140-6736(16)32517-X PMC6886121

[2] Reck M , Rodriguez‐Abreu D , Robinson AG *et al* Pembrolizumab versus chemotherapy for PD‐L1‐positive non‐small‐cell lung cancer. N Engl J Med 2016; 375 (19): 1823–33. 27718847 10.1056/NEJMoa1606774

[3] Borghaei H , Paz‐Ares L , Horn L *et al* Nivolumab versus docetaxel in advanced nonsquamous non‐small‐cell lung cancer. N Engl J Med 2015; 373 (17): 1627–39. 26412456 10.1056/NEJMoa1507643 PMC5705936

[4] Gandhi L , Rodriguez‐Abreu D , Gadgeel S *et al* Pembrolizumab plus chemotherapy in metastatic non‐small‐cell lung cancer. N Engl J Med 2018; 22: 2078–92. 10.1056/NEJMoa1801005 29658856

[5] Socinski MA , Jotte RM , Cappuzzo F *et al* Atezolizumab for first‐line treatment of metastatic nonsquamous NSCLC. N Engl J Med 2018; 378 (24): 2288–301. 29863955 10.1056/NEJMoa1716948

[6] Paz‐Ares L , Luft A , Vicente D *et al* Pembrolizumab plus chemotherapy for squamous non‐small‐cell lung cancer. N Engl J Med 2018; 21: 2040–51. 10.1056/NEJMoa1810865 30280635

[7] Sharma P , Hu‐Lieskovan S , Wargo JA , Ribas A . Primary, adaptive, and acquired resistance to cancer immunotherapy. Cell 2017; 168: 707–23. 28187290 10.1016/j.cell.2017.01.017 PMC5391692

[8] Koyama S , Akbay EA , Li YY *et al* Adaptive resistance to therapeutic PD‐1 blockade is associated with upregulation of alternative immune checkpoints. Nat Commun 2016; 7: 10501. 26883990 10.1038/ncomms10501 PMC4757784

[9] Ricciuti B , Genova C , Bassanelli M *et al* Safety and efficacy of Nivolumab in patients with advanced non‐small‐cell lung cancer treated beyond progression. Clin Lung Cancer 2019; 20: 178–85. 30910574 10.1016/j.cllc.2019.02.001

[10] Gettinger SN , Wurtz A , Goldberg SB *et al* Clinical features and management of acquired resistance to PD‐1 axis inhibitors in 26 patients with advanced non‐small cell lung cancer. J Thorac Oncol 2018; 6: 831–9. 10.1016/j.jtho.2018.03.008 PMC6485248 29578107

[11] Gettinger S , Choi J , Hastings K *et al* Impaired HLA class I antigen processing and presentation as a mechanism of acquired resistance to immune checkpoint inhibitors in lung cancer. Cancer Discov 2017; 7 (12): 1420–35. 29025772 10.1158/2159-8290.CD-17-0593 PMC5718941

[12] Zaretsky JM , Garcia‐Diaz A , Shin DS *et al* Mutations associated with acquired resistance to PD‐1 blockade in melanoma. N Engl J Med 2016; 9: 819–29. 10.1056/NEJMoa1604958 PMC5007206 27433843

[13] Anagnostou V , Smith KN , Forde PM *et al* Evolution of neoantigen landscape during immune checkpoint blockade in non‐small cell lung cancer. Cancer Discov 2017; 7 (3): 264–76. 28031159 10.1158/2159-8290.CD-16-0828 PMC5733805

[14] Pu X , Wu L , Su D *et al* Immunotherapy for non‐small cell lung cancers: Biomarkers for predicting responses and strategies to overcome resistance. BMC Cancer 2018; 1: 1082. 10.1186/s12885-018-4990-5 PMC6225701 30409126

